# Modeling and Analysis of *v_gs_* Characteristics for Upper-Side and Lower-Side Switches at Turn-on Transients for a 1200V/200A Full-SiC Power Module

**DOI:** 10.3390/mi11010005

**Published:** 2019-12-18

**Authors:** Maosheng Zhang, Na Ren, Qing Guo, Xiangwen Zhu, Junming Zhang, Kuang Sheng

**Affiliations:** 1 College of Electrical Engineering, Zhejiang University, Hangzhou 310027, China; zhangmaosheng@zju.edu.cn (M.Z.); ren_na@zju.edu.cn (N.R.); zhuxiangwen@zju.edu.cn (X.Z.); zhangjm@zju.edu.cn (J.Z.); shengk@zju.edu.cn (K.S.); 2 State Key Laboratory of Advanced Power Transmission Technology, Beijing 102209, China

**Keywords:** silicon carbide, power module, negative gate-source voltage spike

## Abstract

In this work, a 1200V/200A full-SiC half-bridge power module was fabricated for high-power high-frequency application, and the characteristics of gate-source voltage ($v_{gs}$) at turn-on transient under different output power was investigated via experiments, modeling, and simulation. Also, the comparison of the $v_{gs}$ characteristics between the upper-side and lower-side was conducted. From experiments, the $v_{gs}$ characteristics show negative spike issue and it becomes severe under higher output power conditions. On the other hand, the upper-side and lower-side show different characteristics, namely, the $v_{gs}$ spike of upper-side is superimposed by a 83.3 MHz high frequency oscillation during the process of $v_{gs}$ being pulled down, while the $v_{gs}$ spike of lower-side contains no oscillation. The mechanisms behind the influence of output power on the $v_{gs}$ spike characteristics and their difference between the upper-side and lower-side were studied via modeling and simulation. Equivalent RLC (resistance-inductance-capacitance) circuit models were proposed and established for the gate driver loops of the upper-side and lower-side based on the internal structure of the power module. With the help of the proposed models, $v_{gs}$ characteristics of the upper-side and lower-side were simulated and compared with the experimental results. The trend of changes in the $v_{gs}$ pulling-down and oscillation amplitude along with the increasing output power from simulation are consistent with that of the experimental results. In addition, different conditions of gate resistance for the SiC power module are compared. Through the comparison between the experiments and simulations, the validity of the proposed equivalent RLC circuit model and the rationality of the analysis about the mechanisms behind the $v_{gs}$ characteristics at turn-on transient for SiC half-bridge power module are confirmed.

## 1. Introduction

Compared with the counterpart of silicon devices, SiC power semiconductor devices can operate at a higher frequency because the switching time of Si-IGBT (silicon based insulated gate bipolar transistor) ranges from 50 ns to 200 ns while it decreases to 10–20 ns for SiC devices [1,2,3,4]. Combined with the parasitic parameters of SiC devices and/or modules, the higher switching frequency and higher allowable negative gate-source voltage as well as the lower threshold voltage for SiC devices can also raise some issues, such as voltage overshoot [5], power loss [6], EMI (electromagnetic interference) emission [7,8,9,10], shoot-through or cross-talk fault [11,12,13,14,15,16], and switching oscillation [17,18,19,20,21,22]. These problems have brought great challenges for adopting SiC devices and/or modules into the mainstream power conversion application with high frequency.

The switching transient issues for a half-bridge power module has been investigated by many researchers. For example, reference [23] analyzed the switching resonance phenomenon that occurred during the process of fully turn-on and turn-off. Reference [24] investigated the influence of parasitic element of a discrete SiC MOSFET device on the switching performance, especially on drain-source voltage ($v_{DS}$) characteristics at turn-on transient. Reference [25,26] investigated the effects of increasing load current/output power on Miller plateau voltage and turn-off transient. It is concluded that the output power has great influence on the characteristics of drain current ($i_{DS}$), drain-source voltage ($v_{DS}$), and gate-source voltage ($v_{gs}$) at turn-off transients.

For the $v_{gs}$ characteristics at switching transient, reference [27] investigated the negative gate-source voltage ($v_{gs}$) spike issue of the upper-side switch and viewed this negative voltage spike as a crosstalk issue. The peers only studied the interaction between the upper-side and lower-side switches, and they put efforts in designing gate driver circuits to alleviate the negative $v_{gs}$ spike issues [28,29]. However, the influence of output power on the $v_{gs}$ characteristics at turn-on transients, and the difference between the $v_{gs}$ characteristics for the upper-side and lower-side switches is not fully studied.

In order to study the influences of output power on the turn-on $v_{gs}$ characteristics for high-power high-frequency application, a 1200V/200A full-SiC high-power module and an inductive load double-pulse test rig were fabricated in this work. The amplitudes of $v_{gs}$ spike and oscillation under different output power conditions were investigated. By building up two different equivalent RLC circuit models of the gate loop path for the upper-side and lower-side, the characteristics of the $v_{gs}$ spike at turn-on transient were compared between upper-side and lower-side; the mechanisms behind these different characteristics were also analyzed with the model.

The paper is organized as follows. Section 2 introduces the experiment setup which includes the developed high-frequency full-SiC power module and a clamped inductive load double pulse test rig. Typical turn-on switching process for a SiC power module is also discussed in this section. Section 3 shows the experimental results of the turn-on $v_{gs}$ characteristics under different output power conditions for both upper-side and lower-side switches. Furthermore, in Section 4, equivalent RLC circuit models of the gate loop path is established for the upper-side and lower-side to analyze the negative $v_{gs}$ spike and oscillation issues in different output power cases. The simulation results are compared to the experimental results. Finally, Section 5 concludes the article.

## 2. Experiment background 

### 2.1. Introduction of the Developed SiC Power Module

**Figure 1:**
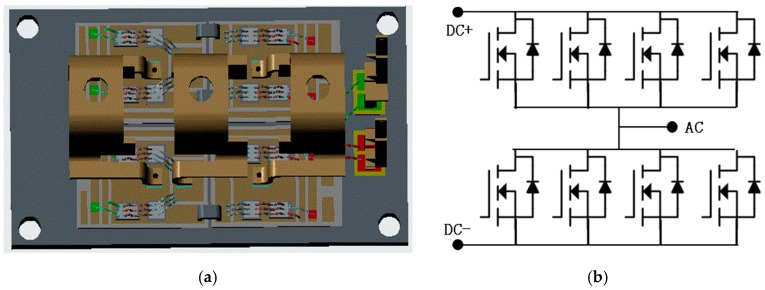
The structure design of the developed 1200V/200A full-SiC power module: (**a**) Design structure; (**b**) circuit topology.

The structure design of the 1200V/200A full-SiC power module with standard package outline is shown in Figure 1a. The diameter of gate wire is 8 mil while other wires inside power module are 14 mil. The footprint of DBC is 34 mm × 25 mm, and the thickness of upper copper, AlN ceramic, and lower copper are 0.3 mm, 0.63 mm, and 0.3 mm, respectively.

The power module has a half-bridge structure topology as shown in Figure 1b, there are four SiC-MOSFET dies (CPM2-1200-0025B, Cree, Inc., Durham, NC, USA) in parallel connection and four SiC-SBD dies (CPW5-1200-Z050B, Cree, Inc., Durham, NC, USA) anti-paralleled with SiC-MOSFETs within each leg of power module. 

This high-power high-frequency SiC power module is developed for the boost converter for hybrid electric vehicles. With an increased switching frequency, the volume of inductor in the boost converter can be reduced, which is desirable for downsizing the power control unit for hybrid electric vehicles. In order to acquire an optimized switching performance in high-power high-frequency application, the design of the power module is optimized from two aspects: One is the symmetry of the structure, the other is the common source inductance.

**Figure 2:**
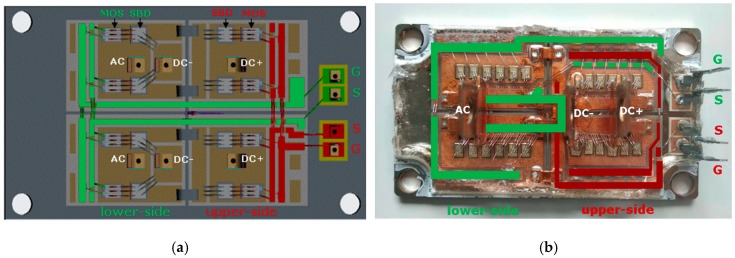
Comparison of common source paths inside the developed SiC power module with the commercial one. (**a**) Developed module in this work (design structure), (**b**) commercial module (physical structure).

For the symmetry design, the two parallel-connected DBCs (direct bonding copper) for both upper-side and lower-side are symmetrical, the two parallel-connected pair of SiC-MOSFET and anti-parallel SiC-SBDs for both upper-side and lower-side are also symmetrical. The symmetry of the gate loops between the parallel chips is realized by placing the gate and source routings of the lower-side near the central line of the module as shown in Figure 2a. When compared to some commercial power module (Figure 2b) where the gate and source routings of the lower-side are placed to one side of the power module, our design has an enhanced symmetry for the gate-source routings of the parallel-connected chips. On the other hand, the parasitic inductance of the gate-source loop of our design is also reduced.

The common source inductance is the parasitic inductance of the common source path of drain routing and gate driver routing inside the power module. Due to its negative feedback effect, a large common source inductance can suppress the change of $v_{gs}$ and slow down the drain current slew rate, which results in a significant increase of switching loss and a decrease of switching frequency [6,24,30,31,32,33]. This means that reducing common source inductance must be considered to speed up the switching of the power module.

In order to minimize the common source inductance, the gate-source routing and drain-source routing are separated in our design, as shown in Figure 2a, where the red lines and green lines stand for the gate driver routing inside the power module for upper-side and lower-side, respectively. The drain routing inside the power module for upper-side starts from DC+ terminal, then goes through MOSFET chips, and crosses SBD chips by wire and copper bridge, and finally goes to AC terminal. For lower-side, the drain routing starts from AC terminal, then goes through MOSFET chips, and crosses SBD chips by wire, and finally goes to DC- terminal (see Figure 2a). There is no common path between the gate-source and drain-source routings inside our power module except the power chips themselves, this means that the common source inductance is minimized in our power module. Thus, switching power loss can be reduced and switching speed can be increased.

### 2.2. Introduction of Double Pulse Testing Platform

**Figure 3:**
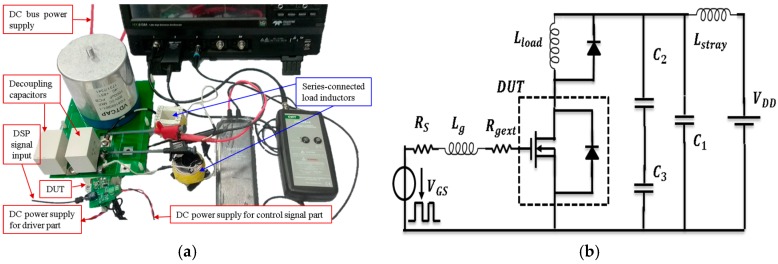
The clamped inductive load double pulse testing circuit platform: (**a**) Physical picture, (**b**) schematic diagram of circuit.

The clamped inductive double-pulse test rig is shown in Figure 3a. The DUT is the developed 1200V/200A full-SiC power module. The electrolytic capacitor of FG810K901-1 from VDTCAP (Shenzhen, China) is used as the DC bus capacitor. Two capacitors from KEMET (Fort Lauderdale, FL, USA) is used as decoupling capacitor. Two serial-connected self-fabricated inductors are used as load inductor with a total inductance of 160 μH. The voltage and current signals are acquired by a Teledyne Lecroy HDO6104 1GHz high definition oscilloscope (Teledyne LeCroy, Chestnut Ridge, New York, USA). A Rogowski current waveform transducer CWT miniHF 1B (Power Electronic Measurements Ltd (PEM), Nottingham, UK) with bandwidth 30 MHz is utilized to measure the drain current of the DUT. A passive voltage probe PP026-2 with bandwidth of 500 MHz and a high voltage differential probe HVD3106 with the bandwidth of 120 MHz from Teledyne Lecroy are used to obtain the waveforms of $v_{gs}$ and $v_{DS}$ of DUT respectively. 

The values of the components in the testing rig are listed in Table 1. Figure 3b shows the schematic diagram of the testing circuit. The output resistance of the gate driver circuit, $R_{S}$, is 0.6 Ω. The gate-source voltage is −2.3 V and 17.5 V for turning off and turning on switches, respectively.

### 2.3. Turn-on Switching Process of SiC Power Module

**Figure 4:**
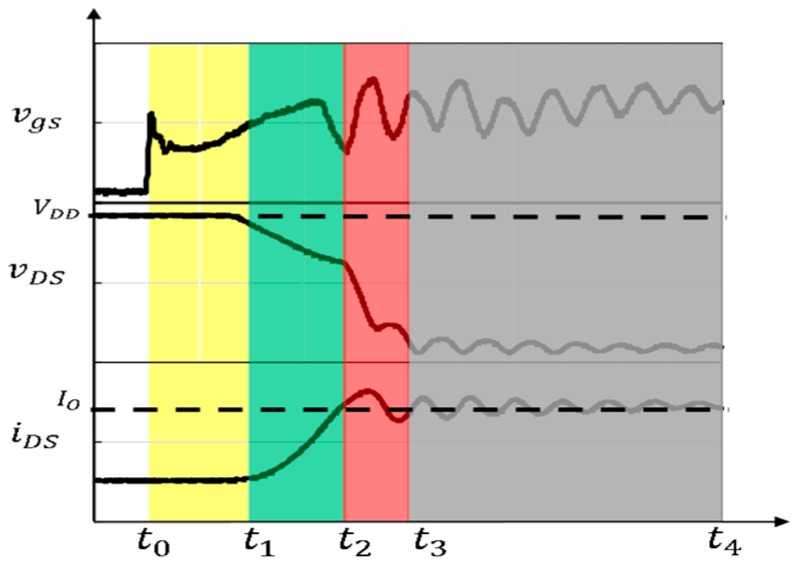
Typical diagram of turn-on switching transient for SiC power module.

The typical turn-on switching transient of SiC power module can be divided by four intervals as shown in Figure 4. 

• Interval 1: From $t_{0}$ to $t_{1}$

The yellow region from $t_{0}$ to $t_{1}$ is the turn-on delay interval, $\tau_{D}$ (on). In this interval the gate voltage charges up the input capacitance of SiC-MOSFETs, the $i_{DS}$ keeps the off-state value until the $v_{gs}$ reaches the threshold voltage, the $v_{DS}$ reduces 10% $\times V_{DD}$ when approaching the end of this period.

• Interval 2: From $t_{1}$ to $t_{2}$

The green region from $t_{1}$ to $t_{2}$ is the turn-on interval 1, $\tau_{1}$ (on). In this interval, the gate voltage continues to charge up the input capacitor of SiC-MOSFETs, the $v_{DS}$ continues to reduce while the $i_{DS}$ rose to $I_{o}$ at the end of this period.

• Interval 3: From $t_{2}$ to $t_{3}$

The red region from $t_{2}$ to $t_{3}$ is the turn-on interval 2, $\tau_{2}$ (on). During the $\tau_{2}$ (on), the gate voltage continues to charge up the input capacitor of SiC-MOSFETs, the $i_{DS}$ continues to change, and the $v_{DS}$ reduces to $V_{DS\left(on\right)}$ with a higher *d*$v_{DS}$*/dt* in this period. The anti-parallel SiC-SBDs begin to regain reverse blocking capability, the rise in voltage across the anti-parallel SiC-SBD causes the SiC-MOSFET voltage to fall rapidly.

• Interval 4: From $t_{3}$ to $t_{4}$

The gray region from $t_{3}$ to $t_{4}$ is used to indicate that the power module is fully turned on, and the time $t_{4}$ is at any moment after $t_{3}$ during turn-on interval. During the fully turn-on interval, all $v_{gs}$, $v_{DS}$, $i_{DS}$ characteristics are kept in oscillation state with their respective frequencies which are determined by the parasitic parameters in the circuit. Due to the different parasitic parameters in drain circuit and gate driver loop, the oscillation frequency of $v_{gs}$ was different from that of $v_{DS}$ and $i_{DS}$.

During the period from $t_{1}$ to $t_{3}$, the increasing drain current $i_{DS}$ makes $v_{DS}$ fall. When $i_{DS}$ is less than $I_{O}$ the freewheeling diodes are forced to conduct current. If the slew rate of $v_{DS}$ is too high before $i_{DS}$ rising to $I_{o}$, the $v_{DS}$ will be limited to a voltage platform. This means the slew rate of $v_{DS}$ is not too high in this sub-interval of $\tau_{1}$ (on). When $i_{DS}$ is increased to be higher than $I_{O}$, the rise in blocking voltage across the anti-parallel SiC-SBD causes the SiC-MOSFET voltage to fall rapidly. This means the slew rate of $v_{DS}$ will be higher in this sub-interval of $\tau_{2}$ (on). Considering the coupling effect of gate-drain capacitor $C_{gd}$ of SiC-MOSFETs inside the power module, the waveform of $v_{gs}$ is affected by the d$v_{DS}/dt$ of drain loop in this period. Meanwhile, the d$v_{DS}/dt$ of drain circuit has greater impacts on the waveform of $v_{gs}$ of gate driver loop during turn-on interval $\tau_{2}$ (on) rather than $\tau_{1}$ (on). It will be discussed in the following section.

## 3. Experimental Results and Comparison

**Figure 5:**
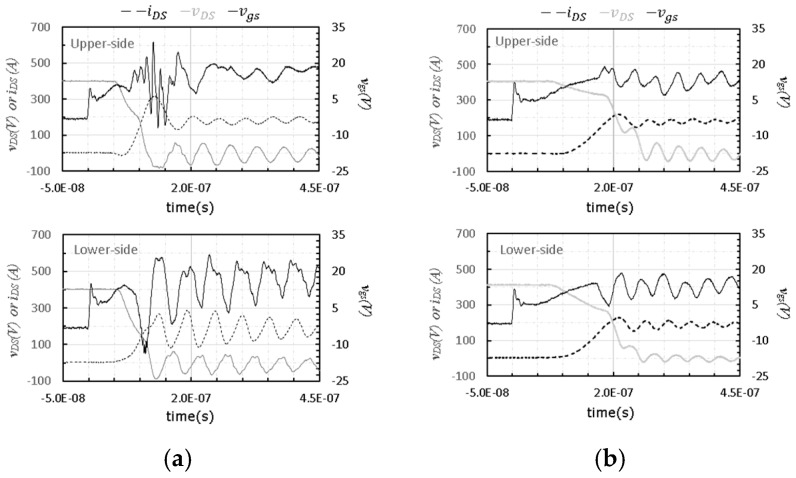
Comparison of transient waveforms between the module developed in this work and one commercial SiC power module ($V_{DD}$ = 400 V, $I_{O}$ = 200 A). (**a**) Developed SiC power module in this work; (**b**) commercial SiC power module.

In order to confirm whether our design can reduce the switching time of the power module, our module and one commercial SiC power module (Figure 2b) were tested based on the built double pulse test platform and gate driver circuit. Their transient waveforms are compared in Figure 5.

As shown in Figure 5, with the same gate driver and double pulse testing platform, the turn-on process of the developed SiC power module was faster than that of the commercial SiC power module. The turn-on times of these two SiC power modules are listed in Table 2. The total turn-on time of the developed module was 110 ns for the upper-side and 117.6 ns for the lower-side. When compared to that of the commercial module, the turn-on time was reduced by 56.4% and 52.0% for the upper-side and lower-side, respectively. Thus, the developed power module is suitable for high frequency application. However, when the turn-on process of the developed power module was speeded up, a severe oscillation and negative voltage spike was observed from the $v_{gs}$ waveforms during the turn-on transient even though the common source inductance was minimized. Therefore, to optimize the high-power high-frequency SiC power module, it is necessary to study the $v_{gs}$ characteristic and find proper designs to address the oscillation and negative voltage spike issues. 

First of all, the factors that take effects on the $v_{gs}$ characteristic will be discussed in the following. It is well known that the increased $V_{DD}$ and $I_{O}$ affects the slew rate of $v_{DS}$. Due to the coupling effects of the drain-gate capacitors, the $v_{gs}$ characteristic will change along with the rise of $V_{DD}$ and $I_{O}$. In order to investigate thoroughly the influences of $V_{DD}$ and $I_{O}$ (i.e., output power) on $v_{gs}$ characteristics at turn-on transient for the developed high frequency SiC power module, and clarify the difference between the $v_{gs}$ characteristics of upper-side and lower-side of the SiC power module, some experiments were conducted. The conditions of these experiments are listed in Table 3. As shown in Table 3, the output power of SiC power module in experiment is increased gradually from case_1 to case_3.

**Figure 6:**
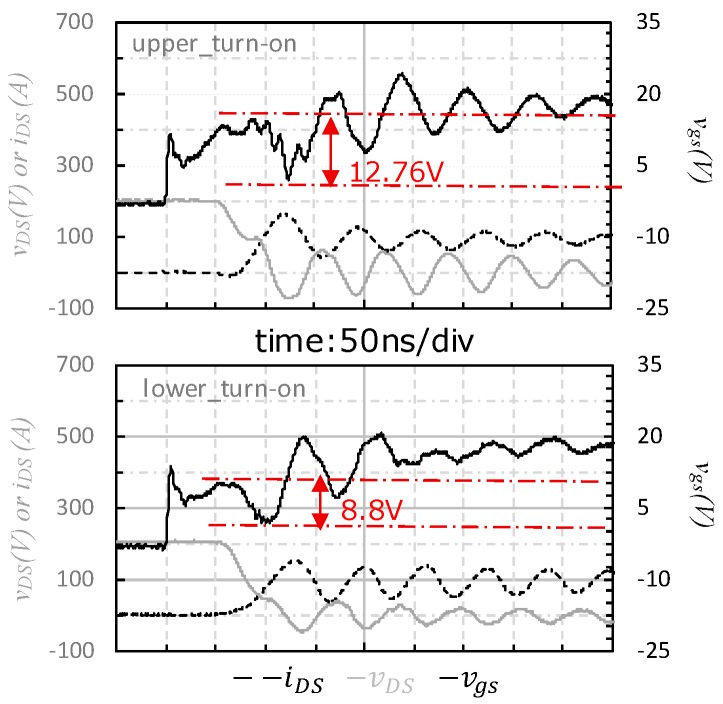
Turn-on transient waveform of case_1.

In the experiment of case_1, the switching waveforms of $v_{gs}$ were almost normal for both upper-side and lower-side though there was minor difference between them as shown in Figure 6. The $v_{gs}$ of upper-side was pulled down by 12.76 V during the $\tau_{2}\left(on\right)$ interval (as shown in Figure 4) while the lower-side’s was pulled down by 8.8 V during the $\tau_{1}\left(on\right)$ interval. The $v_{gs}$ spikes for both sides were kept positive during the turn-on transient, negative $v_{gs}$ spike was absent from the waveforms for both upper-side and lower-side of the SiC power module.

**Figure 7:**
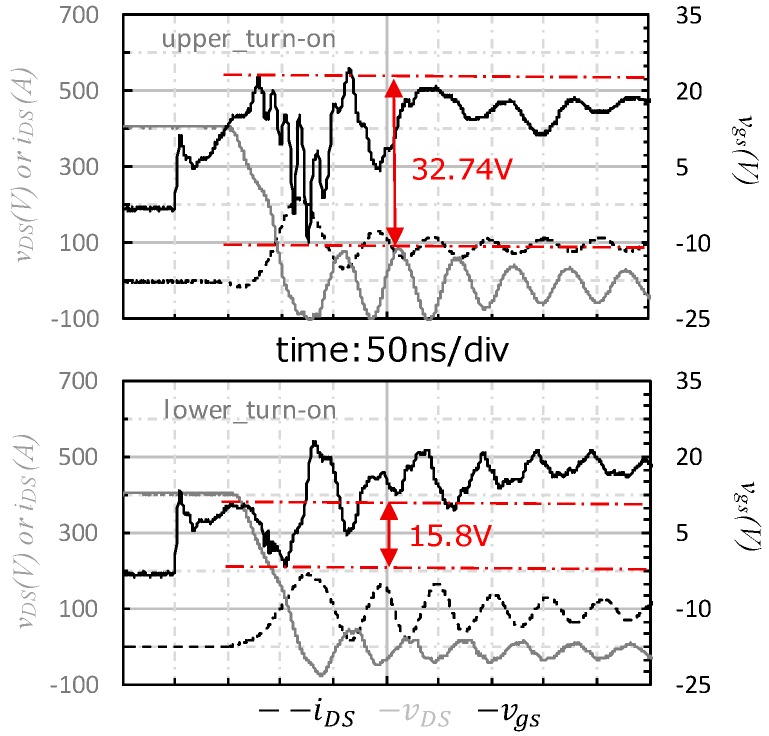
Turn-on transient waveform of case_2.

As output power of the power module rose, the $v_{gs}$ spike started to show different characteristics between the upper-side and lower-side. As shown in Figure 7, the $v_{gs}$ spike of the upper-side was pulled down to an excessively negative voltage (−9.94 V) and accompanied by a significant oscillation with 83.3 MHz frequency during the $\tau_{2}$ (on) interval. On the other hand, the $v_{gs}$ spike of the lower-side was only pulled down to a negative voltage slightly, and no oscillation was observed from the waveform.

As we all know, the oscillation is typically a high frequency signal which put stringent requirements on the bandwidth of probes and the contact quality. In our testing experiment, the bandwidth of the voltage probe was 500 MHz, which is five times the oscillation frequency (83.3 MHz); it is also far greater than the minimum bandwidth requirement for fetching this kind of waveform. In order to eliminate the suspicion that the oscillation was a false waveform, we re-examined and adjusted the connection and contact between the oscilloscope probe and the test point, finding out that oscillation still exists.

**Figure 8:**
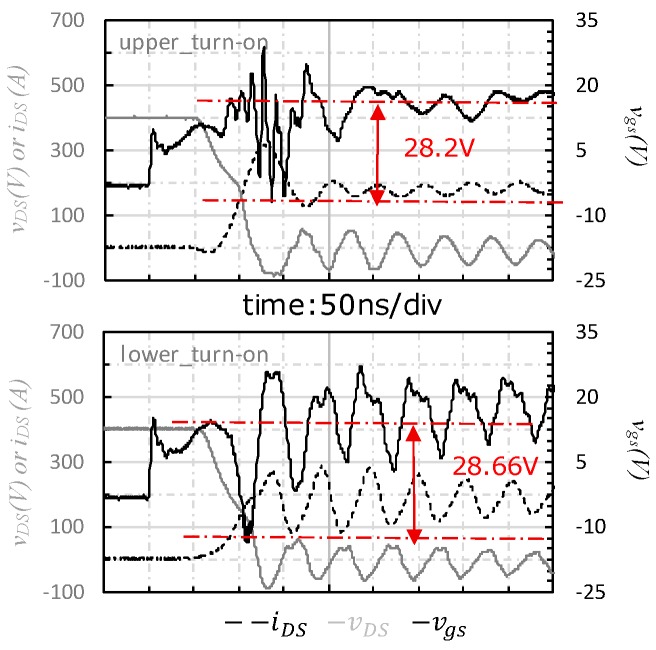
Turn-on transient waveform of case_3.

For the experiment of case_3, the transient waveforms are shown in Figure 8. The $v_{gs}$ spike of the upper-side oscillated more seriously than case_1 and case_2. Both the excessively positive and negative voltage spikes were observed from $v_{gs}$ characteristics of the upper-side, which resulted from the serious oscillation. On the other hand, the $v_{gs}$ of the lower-side was pulled down to a lower negative voltage (−13.7 V), and no oscillation was observed from the waveform.

From the experimental results, it is found that the increase of output power of the SiC power module amplifies the difference of $v_{gs}$ characteristics between upper-side and lower-side. When the output power is relatively low, such as that in case_1, no negative $v_{gs}$ spike appeared; the characteristics of $v_{gs}$ in upper-side are almost the same as that of lower-side. As the output power rises, the negative voltage spike issue of $v_{gs}$ becomes more serious. On the other hand, the difference between the $v_{gs}$ characteristics of the upper-side and lower-side appears. Firstly, the pulling down process of $v_{gs}$ for the upper-side is occurred in the $\tau_{2}\left(on\right)$ interval or the whole period of $v_{DS}$ falling, while that of the lower-side always appears in $\tau_{1}\left(on\right)$ interval. Secondly, the process of $v_{gs}$ pulling down for the upper-side is superimposed by a high frequency oscillation while the lower-side’s is absent from oscillation. Thirdly, the oscillation becomes more serious in upper-side and the negative $v_{gs}$ spike becomes lower in lower-side with an increasing output power (from case_1 to case_3).

**Figure 9:**
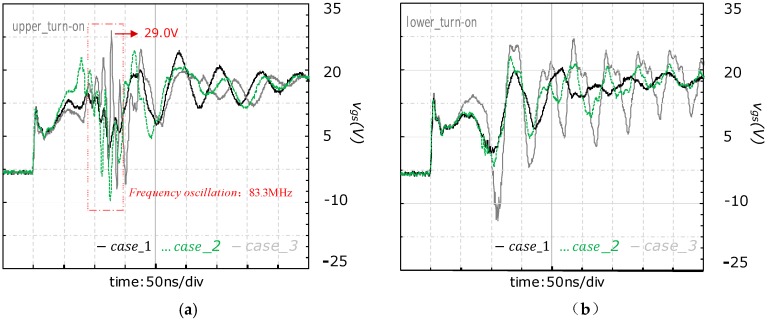
Comparison of turn-on transient waveform of $v_{gs}$ under different output power conditions: (**a**) Turn-on $v_{gs}$ of the upper-side; (**b**) turn-on $v_{gs}$ of the lower-side.

The turn-on transient waveforms of $v_{gs}$ under different output power conditions are compared in Figure 9, where Figure 9a shows the results of the upper-side and Figure 9b shows the results of the lower-side. The detailed information related to the $v_{gs}$ pulling down process are extracted from Figure 9 and summarized in Table 4, such as amplitude of oscillation, voltage of $v_{gs}$ starts to pull down, the lowest $v_{gs}$ spike and the pull-down amplitude.

As shown in Table 4, with an increased output power, the lowest $v_{gs}$ spike continues to decrease, and the amplitude of the $v_{gs}$ pulling down rises significantly for the lower-side. On the other hand, for the upper-side, the pulling down amplitude of $v_{gs}$ in case_3 is smaller than that of case_2. This is because the oscillation of $v_{gs}$ in case_3 is more serious than that of case_2 and the highest $v_{gs}$ spike is up to 29.0 V. These differences of the $v_{gs}$ characteristics between the upper-side and the lower-side is probably attributed to the different *d*$v_{DS}/dt$ of drain loop for the upper-side and lower-side. 

As analyzed in reference [26], the parameters such as gate resistance, gate loop inductance, input capacitance *C_iss_*, and positive gate-source voltage *V_gs_* take effects on the $v_{gs}$ characteristics. In our experiment, the gate driver and testing circuit are the same. This means that the different characteristics of $v_{gs}$ spike between the upper-side and lower-side could be correlated to the different gate-source paths and coupling effects of the d$v_{DS}/dt$ from drain loop to the gate driver loop in the SiC power module.

Meanwhile, we must notice the load current and bus voltage (output power) will influence the switching performance [25]. In other words, even for the same power device, coupling effects between drain loop and gate driver loop by $dv_{DS}/dt$ could be different under different operation conditions. Namely, the different slew rate of $v_{DS}$ during the turn-on transient could bring different extent of coupling influence.

From the experimental results in Figure 6, Figure 7 and Figure 8, the slew rates of $v_{DS}$ at turn-on switching transient can be extracted. The experimental results of $dv_{DS}/dt$ are summarized in Table 5. It is shown that the slew rate of $v_{DS}$ of the upper-side is slightly larger than that of the lower-side, which means the coupling effect of drain loop to gate loop in the upper-side is more significant than that of the lower-side during turn-on transient.

## 4. Modeling and Simulation

### 4.1. Equivalent Model of Gate-Source Path of SiC Power Module

**Figure 10:**
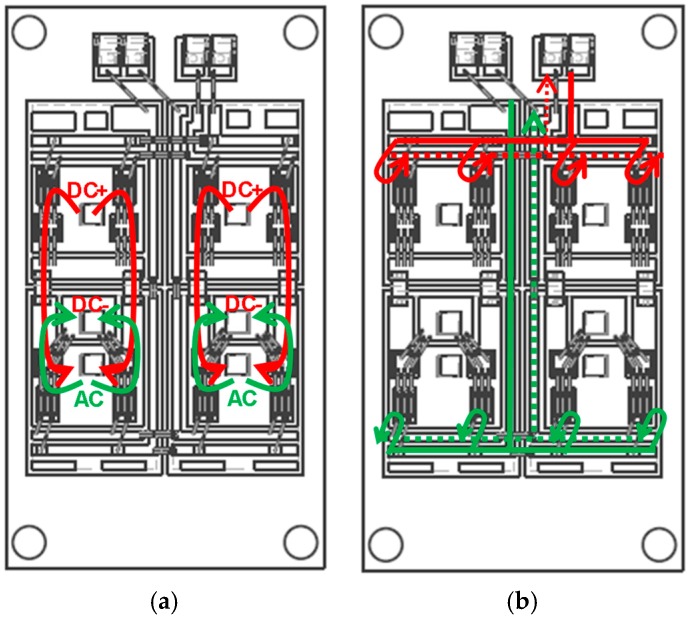
Circuit routings inside the power module (red lines for upper-side; green line for lower-side). (**a**) Drain circuit routings, (**b**) gate loop routings.

The equivalent circuit model of the gate loop is obtained based on the analysis of the actual physical structure of the power module and the output stage of the gate driver circuit. For the standard package outline of the half-bridge module, the internal structure of the upper-side is different from that of the lower-side. The routings of drain circuit and gate circuit inside the power module are shown in Figure 10a,b, respectively. Since all the gate input terminals are placed to the outer edge area of the upper-side of the power module, the gate routings of the lower-side must pass through the upper-side before connecting to the power chips of the lower-side.

Compared with gate-source path of the upper-side, the gate-source path of the lower-side is much longer, the input signal must pass through the whole power module before connecting to the power chips (see Figure 2a, Figure 10). As a result, the location of the lumped parasitic inductance of the gate loop in upper-side is different from that of lower-side in our equivalent circuit models at turn-on transient. That is to say, the topology of the equivalent circuit model of the gate driver loop is different between upper-side and lower-side for the developed half-bridge module.

In the previous analysis of the experimental results, we found that the output power of power module affects the characteristics of $v_{gs}$. As the common source inductance is minimized in our design, the effect of common source inductance on turn-on transient can be ignored. We put emphasis on the coupling effect of drain circuit to gate driver loop, and it will be discussed in the following.

**Figure 11:**
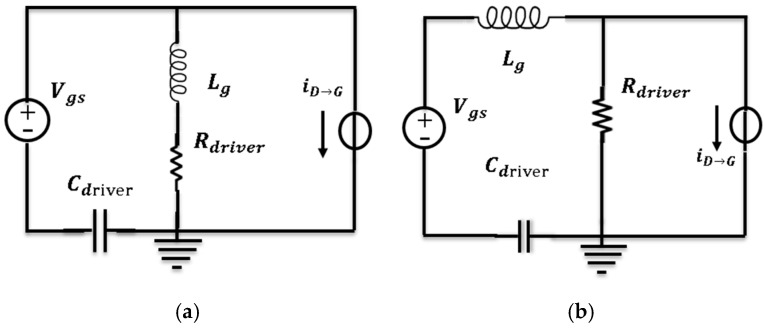
The equivalent circuit models of gate loop path at turn-on transient: (**a**) Upper-side, (**b**) lower-side.

Based on the internal structure of the power module in Figure 10, the equivalent RLC circuit models of the gate driver loop in turn-on transient for the upper-side and lower-side are established and shown in Figure 11a, b, respectively. The coupling effect is equivalent to a short-time current source. As this short-time current source is an external factor for the gate driver loop, it is parallel-connected to the equivalent circuit in the model. With the help of the models, $v_{gs}$ characteristics at the turn-on transient can be simulated. 

In Figure 11, $L_{g}$ stands for the total stray inductance of gate driver loop, it includes the gate-source routings inside module, $L_{gs}$, and the stray inductance of output paths of gate driver circuit, $L_{s}$, which can be expressed by Equation (1),

(1) $$L_{g}=L_{gs}+L_{S}$$

The $R_{driver}$ is the resistance of the whole gate driver loop, it includes the stray resistance of gate-source routings, $R_{gs}$, the external gate resistance $R_{gext}$ and internal resistance of SiC-MOSFET, $R_{gint}$, and the output resistance of gate driver circuit, $R_{s}$, which can be expressed by Equation (2),

(2) $$R_{driver}=R_{gs}+R_{gext}+R_{gint}+R_{S}$$

The $C_{driver}$ is the parasitic capacitance of the total routings of the gate driver loop. The parasitic parameters can be extracted by Q3D software and they are listed in Table 6. 

The value of the equivalent current source stands for the extent of this influence, which is designated as $i_{D\to G}$. The higher the slew rate of $v_{DS}$, the higher the $i_{D\to G}$. The $i_{D\to G}$ is given by Equation (3),

(3) $$i_{D\to G}=C_{gd}\frac{dv_{DS}}{dt}$$

The value of $i_{D\to G}$ is determined by $C_{gd}$ and $\frac{dv_{DS}}{dt}$. The capacitance $C_{gd}$ of power chips increases sharply as the voltage $v_{DS}$ decreases at the turn-on transient, and the value of $i_{D\to G}$ is also increased. Therefore, the coupling effect between the drain loop and gate driver loop is enhanced significantly due to the sharply rising $C_{gd}$ at turn-on transient. 

If the increase in output power is equivalent to a rise of $\frac{dv_{DS}}{dt}$, $i_{D\to G}$ is getting higher at an increased output power. Thus, the current $i_{D\to G}$ can reflect the extent of influence of output power on gate driver loop in our equivalent circuit model. The equivalent current $i_{D\to G}$ at the turn-on transient for the experiment can be calculated by Equation (3) and they are summarized in Table 5.

Based on the RLC circuit model, the extracted parameters of the power module and gate driver circuit, as well as the calculated $i_{D\to G}$ from the experimental results, the generation mechanism of the characteristics of $v_{gs}$ voltage spike for full-SiC power module at turn-on transient can be studied quantitatively by LTspice software. The respective values of $i_{D\to G}$ for case_1, case_2, and case_3 in simulation are the same with those in experiment (Table 5), while the values of the parasitic parameters used in simulation are from Table 6. 

**Figure 12:**
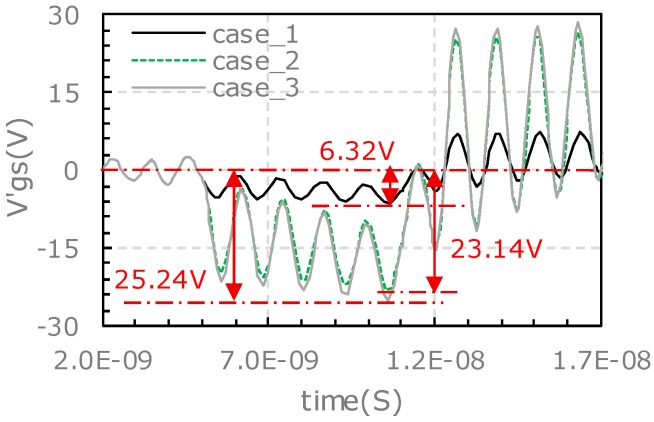
Simulation results of ${v^{\prime }}_{gs}$ voltage spike for upper-side.

For the upper-side, $V_{gs}$ is set to 15 V, the current source $i_{D\to G}$ starts to output pulse at time of 5 ns and it lasts a time interval of 7 ns in the simulation, the value of $i_{D\to G}$ is as listed in Table 5. The simulation results for the three cases as studied by the experiments in Section 3 are shown in Figure 12. 

As the output power increases, the $i_{D\to G}$ rises from 0.47 A to 1.85 A accordingly, the gate-source voltage minus the DC voltage bias (${v^{\prime }}_{gs}$) is pulled down to a lower value. At the meantime, the gate source voltage is accompanied with a high frequency oscillation. The oscillation frequency is 83.3 MHz, which is the same as that of the experimental results. The oscillation frequency doesn’t vary with the $i_{D\to G}$ values as it is only related to the parasitic parameters of both the gate driver circuit and gate-source routings inside the power module. When the equivalent current source starts to output pulse the ${v^{\prime }}_{gs}$ tumbles, and the ${v^{\prime }}_{gs}$ rebounds immediately when the current source output is terminated. 

**Figure 13:**
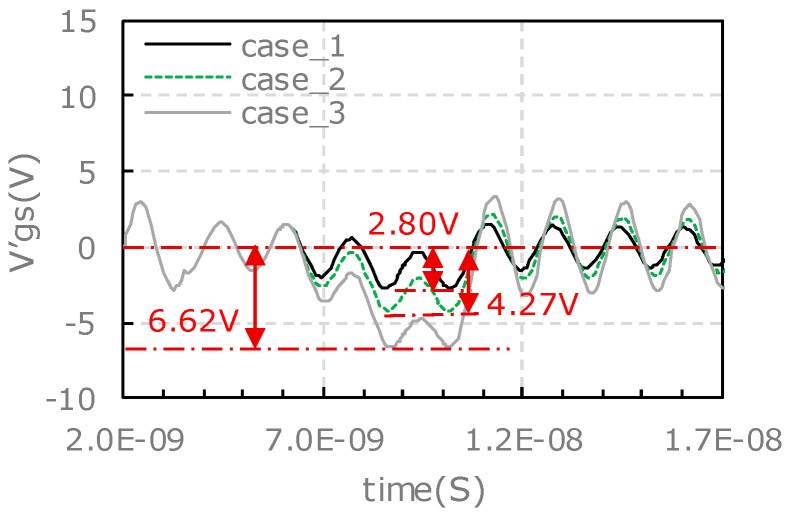
Simulation results of ${v^{\prime }}_{gs}$ voltage spike for lower-side.

For the lower-side, the $V_{gs}$ is set to 15 V, the equivalent current source of $i_{D\to G}$ starts to output pulse at time of 6 ns and it lasts a time interval of 5 ns in the simulation. The simulation results for the three cases as studied by the experiments in Section 3 are shown in Figure 13. As the output power increases, the $i_{D\to G}$ rises from 0.35 A to 1.34 A accordingly, ${v^{\prime }}_{gs}$ is pulled down to a lower value. There is no high frequency oscillation observed.

The characteristics of ${v^{\prime }}_{gs}$ pulling down in simulation are compared with the experimental results (as shown in Figure 9). The amplitude of $v_{gs}$ pulling-down and oscillation for the upper-side and lower-side in different cases are listed in Table 7. For the characteristics of high frequency oscillation, the simulation is in good agreement with the experimental results. For the characteristics of the pulling down amplitude of $v_{gs}$ spike, the simulation is almost in agreement with the experimental results except the upper-side.

The pulling down amplitude of $v_{gs}$ spike of the lower-side increases as the output power rises in both simulation and experiment, but the pulling down amplitude of $v_{gs}$ of the upper-side in simulation is different from the experiment. In simulation, the pulling down amplitude of $v_{gs}$ rises as output increases, but in experimental results the largest pulling down amplitude of $v_{gs}$ occurred in case_2 rather than case_3. This abnormal phenomenon in the experiment is mainly attributed to the high frequency oscillation on the pulling down waveform of $v_{gs}$. As shown in Figure 9a, there is an excessively positive voltage spike of 29.0 V in case_3 during the high frequency oscillation process, while the high frequency oscillation doesn’t bring the voltage spike back to a lower point as that in case_2.

Overall, the characteristics of $v_{gs}$ spike in simulation almost coincide with that of the experiment results. This proves the rationality of our modeling and analysis about the generation mechanism of $v_{gs}$ voltage spike characteristics for SiC power module.

As depicted previously, the negative $v_{gs}$ voltage spike is correlated to the slew rate of $v_{DS}$ and the resistance of the gate driver loop. On one hand, the increased gate resistance can reduce $dv_{DS}/dt$, and decrease the coupling between the drain loop and gate driver loop due to a lower $i_{D\to G}$ at a slower slew rate of $v_{DS}$. On the other hand, the larger the gate resistance of the gate driver loop, the smaller the gate current and voltage spike, and less serious the oscillation at turn-on switching transient. Although the coupling effects are different between the upper-side and lower-side, both the negative $v_{gs}$ spike and high frequency oscillation could shrink as the resistance of the gate driver circuit rises. Another experiment and simulation with a higher external gate resistance are carried out to further verify our RLC circuit model and analysis.

### 4.2. Verification for the Proposed RLC Circuit Model and Analysis

**Figure 14:**
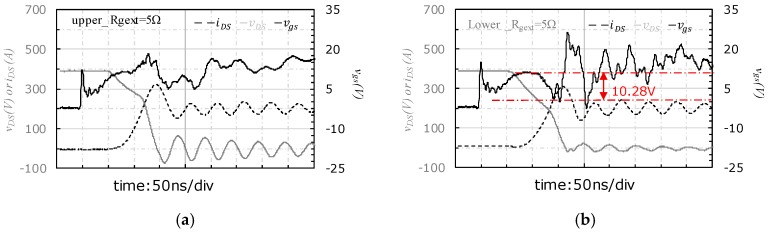
Transient waveform at the condition for output power of case_3 and the external gate resistor equal to 5 Ω: (**a**) Upper-side, (**b**) lower-side.

A resistor of 5 Ω instead of the previous external gate resistor of 2.4 Ω is used in the new experiment. The case_3 is selected for the case study as the output power is highest and the negative spike/oscillation is the most significant in this case. The experimental results are shown in Figure 14.

There is neither voltage pulling down nor high frequency oscillation observed for the upper-side (Figure 14a), but a pulling down effect and a spike of $v_{gs}$ is observed for the lower-side (Figure 14b). When the external gate resistance is increased from 2.4 Ω to 5 Ω, the negative spike of $v_{gs}$ in lower-side is decreased from −13.7 V (in Figure 8 and Table 4) to −2.5 V (Figure 14b). Accordingly, the amplitude of $v_{gs}$ pulling down is reduced from 28.66 V (in Figure 8 and Table 4) to 10.28 V (Figure 14b). 

The experimental results show that the relatively larger external gate resistor can damp the high frequency oscillation in the upper-side and weaken the amplitude of pulling down of $v_{gs}$ at turn-on transient in SiC power module. 

The slew rate of $v_{DS}$ at the turn-on transient are extracted from the experiments and listed in Table 8. The introduced extra current of $i_{D\to G}$ is calculated with Equation (3) and listed in Table 8 as well.

Compared with that of 2.4 Ω, the slew rate of $v_{DS}$ in the condition of gate resistor of 5 Ω reduces significantly; accordingly, the equivalent current $i_{D\to G}$ from drain loop to gate driver loop due to the coupling effect at the turn-on transient also decreases dramatically.

**Figure 15:**
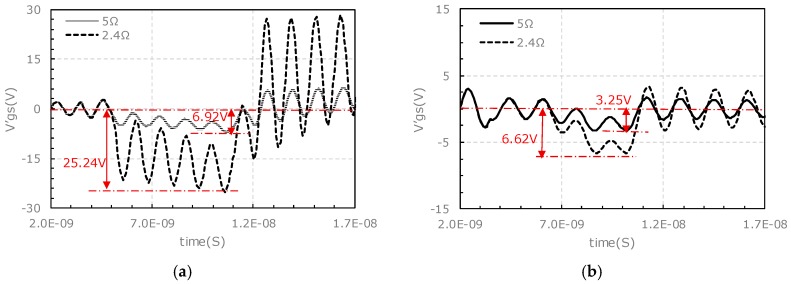
Simulation results of $v_{gs}$ voltage spike for *R_gext_* = 5 Ω and *R_gext_* = 2.4 Ω: (**a**) Upper-side, (**b**) lower-side.

With the proposed equivalent RLC circuit models shown in Figure 11 and Equations (1)–(3), simulation study with an external gate resistor of 5 Ω is carried out. In this simulation all the parameters are the same as that of the previous simulation except the gate resistance. The simulation results under different external gate resistance conditions are compared in Figure 15. For the upper-side, the high frequency oscillation is alleviated by the higher gate resistance (5 Ω vs. 2.4 Ω), and the amplitude of $v_{gs}$ tumbling is reduced from 25.24 V to 6.92 V (72.5% lower). For the lower-side, the amplitude of $v_{gs}$ tumbling is reduced by ~50% from 6.62 V to 3.25 V.

The characteristics from simulation results (in Figure 15) are extracted and compared with those from the experimental results in Figure 8, 14. The compared characteristics contains the amplitude of $v_{gs}$ pulling down and oscillation. The comparison results are listed in Table 9.

As the gate resistance rises, the characteristics of high frequency oscillation in the upper-side disappears for both experiment and simulation, the amplitude of the pulling down of $v_{gs}$ in the upper-side is lowered significantly for both experiment and simulation. The pulling down amplitude in the experiment reduces from 28.20 V to zero while it decreases from 25.24 V to 6.92 V in the simulation. The amplitude of the pulling down of $v_{gs}$ in the lower-side is also reduced significantly for both experiment and simulation, which reduces from 28.66 V to 10.28 V in the experiment and decreases from 6.62 V to 3.25 V in the simulation.

The specific amplitudes of the pulling down of $v_{gs}$ between the simulation and experiment are different, the error could come from the read of d$v_{DS}/dt$ and the parameter extraction by Q3D software. As shown in Table 9, the trend of changes in the amplitude of $v_{gs}$ pulling down and oscillation along with the increase of output power in simulation generally agrees with those of the experimental results. This further confirms the validity of the proposed equivalent RLC circuit model and the rationality of the analysis about the mechanisms behind the $v_{gs}$ characteristics at turn-on transient for the SiC half-bridge power module. 

As guided by the proposed model, the gate driver design must be considered together with the power module design for obtaining an optimized switching performance for the high-power high-frequency SiC power module. For a fabricated power module, we can set the parameters in the model related to the gate driver circuit, such as the $L_{S}$ in Equation (1), the $R_{gext}$ and $R_{S}$ in Equation (2). Then the $v_{gs}$ characteristic can be simulated and optimized by tuning the parameters. Thus, a proper design of the gate driver circuit matched with the power module design and output power level can be obtained in a short design cycle. 

## 5. Conclusions

In this paper, a 1200V/200A full-SiC half-bridge power module was fabricated for high-power high-frequency application. The power module is designed with a symmetrical structure and minimized common source inductance to pursue a faster switching. However, severe oscillation and negative voltage spike issues are observed from the $v_{gs}$ waveforms during the turn-on transient, especially at higher output power level.

The characteristics of $v_{gs}$ at turn-on transient under different output power were investigated and their comparison between the upper-side and lower-side was conducted. From experiments, the $v_{gs}$ characteristics show negative spike issue and it becomes severe under higher output power conditions. On the other hand, the upper-side and lower-side show different characteristics, namely, the $v_{gs}$ spike of upper-side is superimposed by a 83.3 MHz high frequency oscillation during the process of $v_{gs}$ being pulled down, while the $v_{gs}$ spike of lower-side contains no oscillation. 

The mechanisms behind the influence of output power on the $v_{gs}$ characteristics and the difference of $v_{gs}$ characteristics between upper-side and lower-side were studied via modeling and simulation. Equivalent RLC circuit models were proposed and established for the gate driver loop based on the internal structure of the power module. In the models, the coupling effects between drain circuit and gate driver loop is considered and equalized by a current source ($i_{D\to G}$). The value of the equivalent current source is determined by gate-drain capacitance *C_gd_* and $dv_{DS}/dt$. As the increase of output power will contribute to a higher $dv_{DS}/dt$, $i_{D\to G}$ in the model is increased, i.e., the coupling effect between the drain circuit and gate driver loop is enhanced. Thus, the negative $v_{gs}$ spike issue becomes severe in higher output power conditions. On the other hand, when comparing the upper-side and lower-side, the models are different for them as the gate-source path routings are different. Thus, they show different $v_{gs}$ characteristics. And, the higher output power (higher $i_{D\to G}$) will enhance the difference.

With the help of the proposed models, $v_{gs}$ characteristics of the upper-side and lower-side were simulated and compared with the experimental results. The pulling down amplitude of $v_{gs}$ spike in the lower-side increases as the output power rises in both simulation and experiment, as well as the amplitude of the oscillation in the upper-side. Therefore, the trend of changes in the $v_{gs}$ characteristics along with the increasing output power from simulation are consistent with that of the experimental results.

In addition, different conditions of gate resistance for the SiC power module are compared. A higher gate resistance can reduce $dv_{DS}/dt$, thus the $v_{gs}$ spike issue and oscillation can be alleviated. Based on the proposed models, the trend of changes in the $v_{gs}$ characteristics along with the increasing gate resistance can be simulated, and the results are shown to be consistent with that of the experimental results. This further confirms the validity of the proposed equivalent RLC circuit model and the rationality of the analysis about the mechanisms behind the $v_{gs}$ characteristics at turn-on transient for the SiC half-bridge power module. Based on the model, a proper design of the gate driver circuit matched with the power module design and output power level can be obtained in a short design cycle.

## Figures and Tables

**Table 1:** The circuit parameters of components of the testing rig.

Components	Names	Value
DC-bus capacitor	*C*_1_	1000 μF
Decoupling capacitor	*C*_2_	3 μF
Decoupling capacitor	*C*_3_	3 μF
Load inductor	$L_{load}$	160 μH
External gate resistor	*R_gext_*	2.4 Ω

**Table 2:** Comparison of turn-on time for SiC power module.

SiC Module	Commercial Module			Developed Module		
	T_don_ (ns)	T_r_ (ns)	T_total_ (ns)	T_don_ (ns)	T_r_ (ns)	T_total_ (ns)
Upper-side	122.8	129.6	252.4	61.2	48.8	110.0
Lower-side	112.0	133.2	245.2	64.8	52.8	117.6

**Table 3:** $V_{DD}$ and $I_{O}$ parameters for the three cases.

No.	$V_{DD}$ (V)	$I_{O}$ (A)
Case_1	200	100
Case_2	400	100
Case_3	400	200

**Table 4:** Comparison of trainset waveform of $v_{gs}$ at turn-on transient.

Case No.	Location	$v_{gs}$ Value starts to Pull-Down (V)	Amplitude of Oscillation	Lowest $v_{gs}$ Spike (V)	Pull-Down Amplitude (V)
Case_1	Upper-side	14.48	no	1.72	12.76
Case_2	Upper-side	22.80	medium	−9.94	32.74
Case_3	Upper-side	20.44	serious	−7.76	28.20
Case_1	Lower-side	10.32	no	1.52	8.80
Case_2	Lower-side	10.80	no	−4.28	15.08
Case_3	Lower-side	14.96	no	−13.70	28.66

**Table 5:** Comparison of $dv_{DS}/dt$ between upper and lower sides.

Conditions	Upper-Side		Lower-Side	
Case No.	$dv_{DS}/dt$ (V/ns)	$i_{D\to G}$ (A)	$dv_{DS}/dt$ (V/ns)	$i_{D\to G}$ (A)
Case_1	3.92	0.47	2.91	0.35
Case_2	14.25	1.71	6.24	0.75
Case_3	15.4	1.85	11.13	1.34

**Table 6:** Parasitic parameters extracted by Q3D software.

Routings	Symbol	Upper-Side	Lower-Side
Inductance of gate-source routings (nH)	$L_{gs}$	31.50	61.49
Inductance of output path of gate driver circuit (nH)	$L_{s}$	5.00	5.00
Resistance of gate-source routings (Ω)	$R_{gs}$	0.15	0.31
Resistance of output paths of gate driver circuit (Ω)	$R_{S}$	0.60	0.60
Internal resistance of power chips (Ω)	$R_{gint}$	1.00	1.00
Capacitance of gate driver loop (pF)	$C_{driver}$	1.00	1.00

**Table 7:** Comparison of characteristics for simulation and experimental results at turn-on transient.

Characteristics of $v_{gs}$ Pulling Down	Pulling Down Amplitude of $v_{gs}$		Amplitude of High Frequency Oscillation	
	Experiment	Simulation	Experiment	Simulation
Case_1_upper-side	12.76	6.32	tiny	tiny
Case_2_upper-side	32.74	23.14	moderate	moderate
Case_3_upper-side	28.20	25.24	serious	serious
Case_1_lower-side	8.80	2.80	no	no
Case_2_lower-side	15.08	4.27	no	no
Case_3_lower-side	28.66	6.62	no	no

**Table 8:** Comparison of $dv_{DS}/dt$ between the cases with external gate resistance of 5 Ω and 2.4 Ω.

Conditions	Upper-Side		Lower-Side	
*R_gext_*	$dv_{DS}/dt$ (V/ns)	$i_{D\to G}$ (A)	$dv_{DS}/dt$ (V/ns)	$i_{D\to G}$ (A)
2.4 Ω	15.4	1.85	11.13	1.34
5 Ω	3.75	0.45	2.78	0.33

**Table 9:** Comparison of $v_{gs}$ characteristics between experiment and simulation.

Characteristics of $v_{gs}$ Pulling Down	Pulling Down Amplitude of $v_{gs}$ (V)		Amplitude of High Frequency Oscillation	
	Experiment	Simulation	Experiment	Simulation
*R_gext_* = 2.4 Ω_upper-side	28.20	25.24	serious	serious
*R_gext_* = 5 Ω_upper-side	0	6.92	no	no
*R_gext_* = 2.4 Ω_upper-side	28.66	6.62	no	no
*R_gext_* = 5 Ω_upper-side	10.28	3.25	no	no

## References

[1] Josifovic I., Popovic-Gerber J., Ferreira J.A. (2012). Improving SiC JFET Switching Behavior Under Influence of Circuit Parasitics. IEEE Trans. Power Electron..

[2] Wrzecionko B., Bortis D., Biela J., Kolar J.W. (2011). Novel AC-Coupled Gate Driver for Ultrafast Switching of Normally Off SiC JFETs. IEEE Trans. Power Electron..

[3] Lechler M., Piepenbreier B. Regenerative SiC frequency converter with compact Z-source DC-link and sinusoidal output voltage. Proceedings of the International Symposium on Power Electronics, Electrical Drives, Automation and Motion.

[4] Anthony P., McNeill N., Holliday D. High-speed resonant gate driver with controlled peak gate voltage for silicon carbide MOSFETs. Proceedings of the 2012 IEEE Energy Conversion Congress and Exposition (ECCE).

[5] Jauregui D. (2009). Reducing Ringing through PCB Layout Techniques.

[6] Dong Z., Wu X., Sheng K., Zhang J. Impact of common source inductance on switching loss of SiC MOSFET. Proceedings of the 2015 IEEE 2nd International Future Energy Electronics Conference (IFEEC).

[7] Rondon-Pinilla E., Morel F., Vollaire C., Schanen J.L. (2013). Modeling of a Buck Converter with a SiC JFET to Predict EMC Conducted Emissions. IEEE Trans. Power Electron..

[8] Czuchra W., Mysiński W., Woszczyna B. (2016). Electromagnetic compatibility of SiC technology power converter. Tech. Trans..

[9] Rondon E., Morel F., Vollaire C., Schanen J.L. Impact of SiC components on the EMC behaviour of a power electronics converter. Proceedings of the 2012 IEEE Energy Conversion Congress and Exposition (ECCE).

[10] Robutel R., Martin C., Buttay C., Morel H., Mattavelli P., Boroyevich D., Meuret R. (2013). Design and Implementation of Integrated Common Mode Capacitors for SiC-JFET Inverters. IEEE Trans. Power Electron..

[11] Yin S., Tseng K.J., Tong C.F., Simanjorang R., Gajanayake C.J., Gupta A.K. A novel gate assisted circuit to reduce switching loss and eliminate shoot-through in SiC half bridge configuration. Proceedings of the 2016 IEEE Applied Power Electronics Conference and Exposition (APEC).

[12] Yin S., Tseng K.J., Tong C.F., Simanjorang R., Gajanayake C.J., Nawawi A., Liu Y., Liu Y., See K.Y., Sakanova A. Gate driver optimization to mitigate shoot-through in high-speed switching SiC half bridge module. Proceedings of the 2015 IEEE 11th International Conference on Power Electronics and Drive Systems.

[13] Zhang Z., Wang F., Tolbert L.M., Blalock B.J. (2013). Active Gate Driver for Crosstalk Suppression of SiC Devices in a Phase-Leg Configuration. IEEE Trans. Power Electron..

[14] Zhang W., Zhang L., Mao P., Chan X. Analysis of SiC MOSFET Switching Performance and Driving Circuit. Proceedings of the 2018 IEEE International Power Electronics and Application Conference and Exposition (PEAC).

[15] Li H., Zhong Y., Yu R., Yao R., Long H., Wang X., Huang Z. (2019). Assist Gate Driver Circuit on Crosstalk Suppression for SiC MOSFET Bridge Configuration. IEEE J. Emerg. Sel. Top. Power Electron..

[16] Zhang W., Zhang Z.Y., Wang F., Costinett D., Tolbert L., Blalock R. Common source inductance introduced self-turn-on in MOSFET turn-off transient. Proceedings of the 2017 IEEE Applied Power Electronics Conference and Exposition (APEC).

[17] Lemmon A., Mazzola M., Gafford J. (2014). Instability in half-bridge circuits switched with wide band-gap transistors’. IEEE. Trans. Power Electron..

[18] Alatise O., Parker-Allotey N.A., Hamilton D., Mawby P. (2012). The Impact of Parasitic Inductance on the Performance of Silicon–Carbide Schottky Barrier Diodes. IEEE Trans. Power Electron..

[19] Yang F., Wang Z.J., Zhang Z., Campbell S., Wang F., Chinthavali M. Understanding middle-point inductance’s effect on switching transients for multi-chip SiC package design with P-cell/N-cell concept. Proceedings of the 2018 IEEE Applied Power Electronics Conference and Exposition (APEC).

[20] Liu T., Wong T.T.Y., Shen Z.J. (2019). A Survey on Switching Oscillations in Power Converters. IEEE J. Emerg. Sel. Top. Power Electron..

[21] Middelstaedt L., Lindemann A. Optimization of critical oscillations within a boost converter based on an analytical model. Proceedings of the 2016 18th European Conference on Power Electronics and Applications (EPE’16 ECCE Europe).

[22] Jahdi S., Alatise O., Alexakis P., Ran L., Mawby P. (2014). The Impact of Temperature and Switching Rate on the Dynamic Characteristics of Silicon Carbide Schottky Barrier Diodes and MOSFETs. IEEE Trans. Ind. Electron..

[23] Liu T., Ning R., Wong T.T.Y., Shen Z.J. (2016). Modeling and Analysis of SiC MOSFET Switching Oscillations. IEEE J. Emerg. Sel. Top. Power Electron..

[24] Wang J., Chung H.S.H., Li R.T.H. (2012). Characterization and Experimental Assessment of the Effects of Parasitic Elements on the MOSFET Switching Performance. IEEE Trans. Power Electron..

[25] Zhao S., Dearien A., Wu Y., Farnell C., Rashid A.U., Luo F., Mantooth H.A. (2019). Adaptive Multi-Level Active Gate Drivers for SiC Power Devices. IEEE Trans. Power Electron..

[26] Lobsiger Y., Kolar J.W. (2015). Closed-loop di/dt and dv/dt IGBT gate driver. IEEE Trans. Power Electron..

[27] Zhang B.F., Xu J.M., Qian Q., Zhang Z., Xie S.J. (2016). Analysis on Characteristics of SiC-MOSFET and Key Techniques of Its Applications. J. Power Supply.

[28] Zhou Q., Gao F. (2018). A gate driver of SiC MOSFET for suppressing the negative voltage spikes in a bridge circuit. IEEE Trans. Power Electron..

[29] Zeng Z., Li X. (2018). Comparative Study on Multiple Degrees of Freedom of Gate Drivers for Transient Behavior Regulation of SiC MOSFET. IEEE Trans. Power Electron..

[30] Chen Z., Boroyevich D., Burgos R. Experimental parametric study of the parasitic inductance influence on MOSFET switching characteristics. Proceedings of the 2010 International Power Electronics Conference—ECCE ASIA-.

[31] Wang Z.H., Zhang J.M., Wu X.K. Analysis of stray inductance’s influence on SiC MOSFET switching performance. Proceedings of the Energy Conversion Congress and Exposition (ECCE).

[32] Ke J.J., Zhao Z.B., Wei C.J., Xu P., Xie Z.K., Yang F. (2017). Effect of the Parasitic Inductance on SiC MOSFET Switching Characteristics. Semicond. Technol..

[33] Yang B., Zhang J. Effect and utilization of common source inductance in synchronous rectification. Proceedings of the Twentieth Annual IEEE Applied Power Electronics Conference and Exposition, APEC 2005.

